# The Effects of Ghrelin on Energy Balance and Psychomotor Activity in a Goldfish Model: An Overview

**DOI:** 10.1155/2011/171034

**Published:** 2011-05-24

**Authors:** Ki Sung Kang, Satowa Yahashi, Kouhei Matsuda

**Affiliations:** Laboratory of Regulatory Biology, Graduate School of Science and Engineering, University of Toyama, 3190-Gofuku, Toyama 930-8555, Japan

## Abstract

The goldfish (*Carassius auratus*) has a number of merits as a laboratory animal, and we have extensively identified the mechanisms by which ghrelin regulates food intake in this species. For the first time, we have purified and characterized 11 molecular variants of ghrelin that are present in goldfish intestine and shown that 17-residue ghrelin, the predominant form with *n*-octanoyl modification, is biologically active and implicated in the regulation of food intake as an endogenous orexigenic factor. Ghrelin and its receptor system are present not only in peripheral tissues such as stomach and intestine, but also in the central nervous system. Recent studies have also revealed that a number of neuropeptides are widely distributed in the brain in key areas of emotional regulation, and their role as modulators of behavioral states is being increasingly recognized. Interestingly, administration of ghrelin induces an orexigenic effect and also modifies locomotor activity, suggesting the involvement of ghrelin in feeding control and regulation of energy balance. Information derived from studies of ghrelin has been increasing, and important results have been obtained from both fish and mammals. Here, we present an overview of the effects of ghrelin on energy balance and psychomotor activity in the goldfish as an animal model. The available data provide an insight into evolutionary background of ghrelin's multiple actions on energy homeostasis in vertebrates.

## 1. Introduction

The goldfish (*Carassius auratus*) has been employed as a useful laboratory animal in the fields of cell biology, immunology, endocrinology, molecular evolution and comparative genomics, neurobiology, and learning and memory [1–7]. The goldfish has several merits as a laboratory animal. Under experimental conditions, goldfish can be entrained to feed at a specific time of day on a diet sufficient to maintain a high body growth rate and have a constant daily food intake [8]. The basic mechanisms responsible for control of feeding behavior are conserved across vertebrates from fish to mammals [9]. Food intake in the goldfish can be measured by directly observing and recording the number of food pellets eaten by an individual fish [8]. Peptides or peptide analogs can be easily injected into the third ventricle of the brain, thus providing highly valuable information on feeding behavior [10]. We and others have extensively identified the regulatory mechanisms mediated by neuropeptides, such as that of ghrelin on food intake, using goldfish models [3, 10–15]. For example, acyl ghrelin, neuropeptide Y (NPY), and orexin-A exert orexigenic effects, while corticotropin-releasing hormone (CRH), *α*-melanocyte-stimulating hormone (*α*-MSH), and cholecystokinin (CCK) suppress appetite in this species (reviewed in [15]).

Ghrelin, a 28-amino-acid residue peptide that was first isolated from rat and human stomachs, acts as an endogenous ligand for the growth hormone secretagogues (GHSs) receptor. GHSs act at the GHS receptor to stimulate the release of growth hormone (GH) in mammals as well as fish [10, 16–21]. The GHS receptor is a member of the G protein-coupled receptor superfamily with seven transmembrane domains [22]. Ghrelin possesses an *n*-octanoic acid modification at the third N-terminal serine residue, which is essential for its biological activity in mammals [17]. The primary structure of ghrelin has been determined not only in mammals, but also in teleost fish species including the goldfish, Japanese eel (*Anguilla japonica*), rainbow trout (*Oncorhynchus mykiss*), tilapia (*Oreochromis mossambicus*), and channel catfish (*Ictalurus punctatus*) [18, 19, 23–27]. The fish ghrelins that have been characterized so far possess an amidated C-terminus, except for channel catfish ghrelin, which has 23 amino acid residues with a free terminus [18, 19, 25, 26]. However, the structure of goldfish ghrelin used in earlier studies was deduced from its precursor cDNA sequence, and the native form of goldfish ghrelin had yet to be identified in the goldfish itself. In several species including humans and fish, the existence of ghrelin with different fatty acid modifications has been reported [18, 19, 25, 26, 28]. For the first time, we have purified and characterized 11 molecular variants of ghrelin that are present in goldfish intestine and shown that 17-residue ghrelin, the predominant form with an n-octanoyl modification, is biologically active and implicated in the regulation of food intake as an endogenous orexigenic factor in this species [27]. The C-terminus of almost all the goldfish ghrelin isolated was not amidated although other teleost ghrelins identified so far have the amide structure at the C-terminus as described above [18, 19, 25, 26].

Ghrelin mRNA is particularly abundant in the stomach and intestine and is also expressed at low levels in other organs, notably the brain (mainly the diencephalon), pituitary, heart, lung, pancreas, kidney, and placenta [17, 29–31]. The GHS receptor is also present in the brain, pituitary, gastrointestinal tract, kidney, pancreas, and heart [22, 32]. Ghrelin is now considered to be a multifunctional peptide involved in the regulation of food intake and energy homeostasis in mammals and nonmammalian vertebrates [26, 33].

## 2. Effect of Ghrelin on Energy Balance in Goldfish

The regulation of energy balance is related to somatic growth and instinctive behavior, including feeding, reproduction, and emotion, and is a complex phenomenon involving interaction of the central and peripheral nervous systems, neuroendocrine system and gastrointestinal system [34, 35]. The hypothalamic region of the brain plays an important role in the regulation of feeding and neuroendocrine functions [36]. Many types of neurons in the hypothalamus and related regions express neuropeptides, such as ghrelin [17], orexin [37], NPY [38], agouti-related peptide (AGRP) [36], melanin-concentrating hormone (MCH) [39], proopiomelanocortin-derived peptides [40], pituitary adenylate cyclase-activating polypeptide (PACAP) [41, 42], CRH [43], neuromedin U (NMU) [44], and diazepam-binding inhibitor- (DBI-) derived peptides [45, 46], which have all been implicated in the regulation of feeding behavior and also energy homeostasis in mammals.

**Figure 1:**
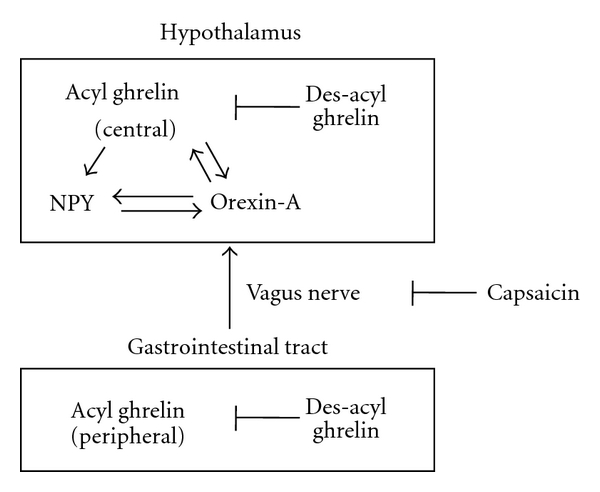
Schematic drawing of the orexigenic signaling pathways mediated by ghrelin in the peripheral and central nervous system in the goldfish.

Ghrelin plays an important role in energy balance by regulating food intake, body weight, and glucose homeostasis [47–49]. We have isolated the native form of goldfish ghrelin from the intestine [27], and it has been shown that intracerebroventricular (ICV) and intraperitoneal (IP) injection of goldfish ghrelin with an *n*-octanoic acid (acyl) moiety at the third serine residue stimulates food intake as well as the release of GH and gonadotropins from the pituitary gland [10, 27]. The orexigenic action of ghrelin is blocked by treatment with NPY Y1 receptor and orexin receptor-1 antagonists in goldfish [50, 51], suggesting that this action is mediated by the NPY Y1 receptor- and orexin receptor-1-signaling pathways. Our previous data have also indicated that in goldfish, ICV injection of MCH inhibits food intake and decreases the expression of mRNA for ghrelin in the hypothalamus [52], suggesting that ghrelin and MCH functionally interact or exhibit mutual inhibitory effect to regulate feeding behavior in teleost fish. In contrast, ICV and IP administration of des-*n*-octanoylated (des-acyl) ghrelin produces no changes in orexigenic activity in goldfish [13, 14]. This had been interpreted to indicate that des-acyl ghrelin has no effect on food intake. Surprisingly, however, the ICV and IP injection of des-acyl ghrelin at doses 3–10 times higher than that of acyl ghrelin was later shown to suppress the orexigenic action of ICV- and IP-injected acyl ghrelin, suggesting that des-acyl ghrelin functionally inhibits acyl ghrelin-induced orexigenic activity in goldfish [13]. A recent study has also revealed that similarly, des-acyl ghrelin suppresses acyl ghrelin-induced appetite in rats [53], suggesting that des-acyl ghrelin is involved in the regulation of appetite in vertebrates [35] (Figure 1). The vagus nerve has primary neuroanatomic roles in the gut-brain axis, transmitting meal-related signals elicited by contact of nutrient with the gastrointestinal tract to sites in the central nervous system that mediate ingestive behavior [54]. We previously examined the effect of capsaicin, a neurotoxin which destroys vagal afferents, on the orexigenic activity induced by IP injected acyl ghrelin [13]. Pretreatment with IP-injected capsaicin cancelled the orexigenic action of IP-injected acyl ghrelin in goldfish (Figure 1).

**Figure 2:**
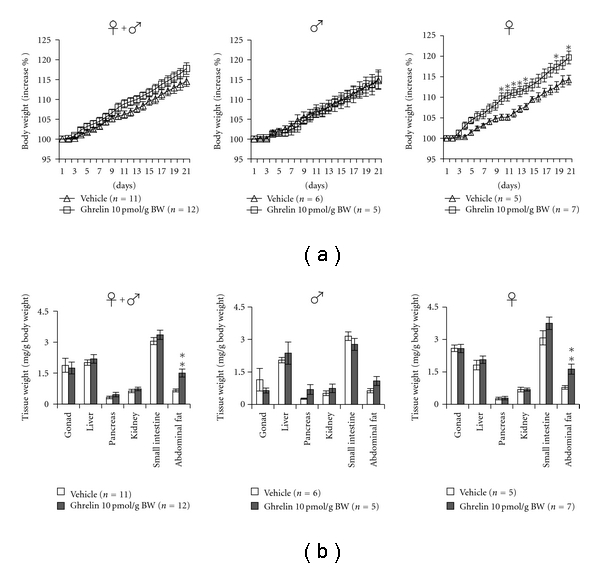
Effect of long-term IP injection of ghrelin on body and organ weights. Panel (a) shows the changes in body weight of goldfish during the experimental period. Panel (b) shows the changes in organ weight of goldfish after 21 consecutive days of IP ghrelin injection. Each column and bar represent the mean and S.E.M., respectively, and the numbers in parentheses in the legend indicate the number of fish used in each group. The significance of differences at each time point, compared to the vehicle-injected group, was evaluated by one-way ANOVA with Bonferroni's method (*P < .05, **P < .01).

Recently, it has been demonstrated that ghrelin plays an important role in the regulation of central and peripheral lipid metabolism through specific control of hypothalamic AMP-activated protein kinase (AMPK), a critical metabolic regulator of both cellular and whole-body energy homeostasis [55, 56]. We have conducted IP injection of ghrelin for 21 consecutive days to identify its long-term effects on growth and lipid metabolism in goldfish. The average body weight of goldfish (both sexes) increased gradually in the vehicle-treated group but increased slightly after ghrelin (10 pmol/g BW) cotreatment. However, when body weight was examined according to sex, female goldfish showed a significant increase of body weight after ghrelin cotreatment, whereas male goldfish showed no change (Figure 2(a)). Interestingly, a comparison of tissue weights (gonad, liver, pancreas, kidney, small intestine, and abdominal fat) showed that the amount of abdominal fat in female goldfish was significantly increased after 21 consecutive days of ghrelin treatment (Figure 2(b)). The level of lipid deposition in liver tissue of female goldfish was also significantly increased after IP injection of ghrelin for 21 consecutive days [34], in line with the observation that ghrelin induces abdominal obesity via lipid retention in rats [57]. Therefore, there is a sex difference in the effect of ghrelin on lipid metabolism in goldfish although the mechanism involved is still unknown. As ghrelin induces hepatic steatosis, increasing the number of lipid droplets and the content of triacylglycerol through a GHS receptor-dependent mechanism in rats [57], the lipid accumulation in goldfish may also mediated via a GH and its receptor-related pathways.

## 3. Effect of Ghrelin on Psychomotor Activity in the Goldfish

Recent studies have revealed that neuropeptides are widely distributed in the brain in key areas of emotional regulation and are being increasingly recognized as modulators of behavioral states [34, 58, 59]. In rodents, psychomotor activity and/or emotional behavior are affected by neuropeptides involved in the regulation of feeding, such as ghrelin, orexin, NPY, MCH, *α*-MSH, PACAP, CRH, NMU, 26RFamide, and DBI-derived peptides, suggesting that these neuropeptides play psychophysiological roles, including regulation of feeding and emotion [59–63]. Recent advances in analytical technology and increased interest in fish behavior have revealed that several of these neuropeptides also influence the locomotor or psychomotor activity of teleost fish [34, 58].

**Figure 3:**
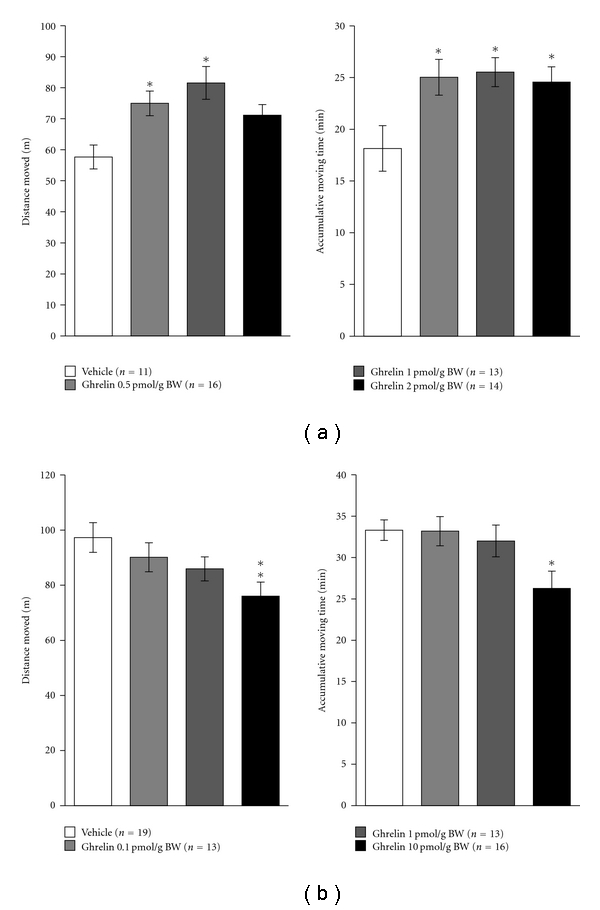
Effect of ghrelin on locomotor activity in goldfish. Panel (a) shows the effect of a single ICV injection of ghrelin on locomotor activity. Panel (b) shows the effect of IP-injected ghrelin on locomotor activity. The open field test, which started 15 min after ICV or IP injection and lasted 45 min, was performed with a video tracking system for automatic recording of goldfish behavior (EthoVision Pro, Noldus Information Technology, Wageningen, Netherlands). Each column and bar represent the mean and S.E.M., respectively, and the numbers in parentheses in the legend indicate the number of fish used in each group. The significance of differences at each time point, compared to the vehicle-injected group, was evaluated by one-way ANOVA with Bonferroni's method (*P < .05, **P < .01).

Low physical activity levels are a major determinant of body fat gain during overfeeding [64]. Locomotion is a form of animal behavior necessary for seeking food, shelter and mates, interaction with competitors, and avoidance of predators [65]. In animal models, locomotor activity is one behavioral measure commonly used to study the level of not only physical activity but also anxiety [66–68]. Tang-Christensen et al. [67] showed for the first time that central administration of ghrelin decreases spontaneous locomotor activity in rats, whereas peripheral ghrelin injections is known to increase locomotor activity of rats [69]. In our study of goldfish [14], ICV injection of ghrelin increased locomotor activity, whereas IP injection of ghrelin at a dose 5 times higher than that used for ICV injection decreased locomotor activity (Figure 3). These results suggest that the central and peripheral actions of ghrelin are involved in the control of psychophysiological functions in rodents as well as in teleost fish although the mechanisms responsible for its different actions among the same or different species are still unclear. Plausible mechanistic pathways have been suggested by studies using rodent models. Ghrelin may mediate locomotor activity via noradrenergic pathways through the interaction with orexin. Dopamine has been implicated in the regulation of appetite and body weight [70, 71] and is certainly important for the central nervous control of motor activity [72, 73]. Involvement of the ventral tegmental area dopaminergic system in orexin-induced activity has been demonstrated [74]. In addition, ghrelin-immunoreactive axonal terminals make direct synaptic contacts with orexin-producing neurons [75], and the orexinergic system is anatomically well placed to influence the arousal, motivational, metabolic, autonomic, and motor processes necessary for elicitting homeostatically appropriate behavior [68]. Therefore, the regulation of locomotor activity by ghrelin in goldfish may also be mediated through interaction with orexin.

**Figure 4:**
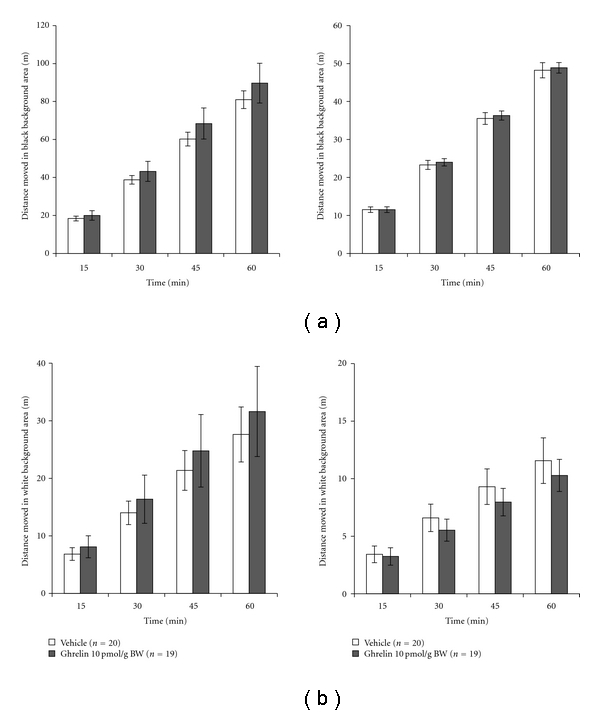
Effect of a single IP injection of ghrelin on black/white background preference in goldfish. Panel (a) shows the effect of ghrelin on the distance moved and latency period of goldfish in the black background area. Panel (b) shows the effect of ghrelin on the distance moved and latency period of goldfish in the white background area. Each column and bar represent the mean and S.E.M., respectively, and the numbers in parentheses in the legend indicate the number of fish used in each group. The data were evaluated by one-way ANOVA with Bonferroni's method.

The scototaxis protocol, a white/black background areas preference test, has recently been employed to investigate the psychomotor activity of fish, notably anxiety-like behavior [76, 77]. In our study with goldfish, intact fish were found to prefer the black to the white background area: the average time spent in the black background compartment was 3 times greater than that in the white background area. This scototaxis test appears to be suitable for evaluating the effect of pharmacological compounds on psychomotor activity in fish [34, 58]. In our experiment involving IP injection of ghrelin in goldfish, there were no significant changes in the time or locomotor activity in the black and white background areas (Figures 4(a) and 4(b)). These results suggest that ghrelin has no effect on anxiety in goldfish. However, several rodent studies have suggested that ghrelin mediates some of the usual behavioral responses to acute and chronic stress [78]. Gastric ghrelin mRNA or total plasma ghrelin increases in response to various forms of acute stress in mouse and rat models [79, 80]. However, another group has demonstrated that ICV administration of ghrelin into the hippocampus, amygdala, or dorsal raphe nucleus induces anxiety-like behaviors in certain rat strains [81]. The reasons for the varied anxiety-related behavioral responses elicited by ghrelin are currently unclear, but differences in experimental conditions, strains, or species have been suggested [78]. Ghrelin-induced orexigenic action is mediated by the NPY Y1 or Y5 receptor-signaling pathway as described above. Recent studies have revealed that NPY also exerts an anxiogenic action via the NPY Y2 or Y4 receptor system [82–86]. It can also be speculated that ICV-injected ghrelin induces anxiety through the NPY Y4 receptor-signaling pathway.

## 4. Conclusion and Perspectives

The goldfish provides an excellent and widely used animal model for studying the neuroendocrine control of feeding behavior [3, 15, 34]. For nonmammals, such as teleost fish, information derived from studies of ghrelin has been increasing, and important results have been obtained. The most significant scientific advances in this respect are largely related to neuroendocrine signaling for regulation of growth, feeding, and psychophysiological behavior [15, 34, 35]. In this paper, we have summarized the effects of ghrelin on energy balance and psychomotor activity in the goldfish and shown that ghrelin plays an important role in energy balance in this species by regulating food intake, lipid metabolism, and also locomotor activity. The findings so far suggest that ghrelin plays a pleiotropic role in the regulations of energy homeostasis in fish through a mechanism similar to that in mammals although some of its actions differ among the same and different species.
